# Complications in the treatment of oropharyngeal carcinoma in patients with systemic sclerosis: A case report

**DOI:** 10.3892/ol.2014.2627

**Published:** 2014-10-22

**Authors:** ALES ČOČEK, ALES HAHN, MILOSLAV AMBRUŠ, MARIE VALEŠOVÁ

**Affiliations:** 1 ENT Clinic, Charles University Third Medical School and Královské Vinohrady Teaching Hospital, 100 34 Prague 10, Czech Republic; 2 Radiotherapy and Oncology Clinic, Charles University Third Medical School and Královské Vinohrady Teaching Hospital, 100 34 Prague 10, Czech Republic; 3 First Internal Medicine Clinic, Charles University Third Medical School and Královské Vinohrady Teaching Hospital, 100 34 Prague 10, Czech Republic

**Keywords:** oropharyngeal carcinoma, systemic sclerosis, autoimmune diseases

## Abstract

Systemic sclerosis is a chronic, progressive disease with an extremely poor prognosis. The incidence of malignant tumors in patients with systemic sclerosis is increased when compared with that of the general population. In certain malignancies, systemic sclerosis presents as a paraneoplastic process. The symptoms of sclerosis in the organs of the head and neck often overlap with symptoms of malignant diseases, which may increase the difficulty of a differential diagnosis. Additionally, the presence of sclerosis may complicate standard examination procedures, due to poor access to the oral cavity and oropharynx. When considering treatment options, it is important to evaluate the surgical and oncological risks to soft tissues of the head and neck with regard to both diseases, as well as the relatively poor prognosis for systemic sclerosis and oropharyngeal cancer. The low incidence of patients with systemic sclerosis and oropharyngeal carcinoma together presents a clear case for a casuistic approach. Based upon our own experience, we can attest to the difficulty of treating such patients. However, we have no evidence to indicate that these patients have reduced tolerance to surgical treatments. The current study presents the case of a 47-year-old female with systemic sclerosis, who was diagnosed with oropharyngeal carcinoma. The patient initially tolerated radiotherapy treatment well, however post-radiotherapy complications occurred. Despite many enigmatic indications to the contrary, it appears that the complications in this instance may be due to late toxicity from radiotherapy.

## Introduction

Systemic sclerosis is a diffuse connective tissue disorder, which affects the skin and internal organs. Its pathological-anatomical basis is the fibrotic sclerotization of the peripheral and internal organ blood vessels. The disease has a chronic, progressive nature and primarily affects females. The skin of the fingers is often the first area to be affected, which may present with atrophy and edema. The skin of the fingers progressively thickens (sclerodactyly) and the skin on the fingers and other parts of the body becomes stiff and shiny. It is also typical for patients to present with a mask-like face, limited facial expressions and perioral furrows. Vascular afflictions manifest as Raynaud’s phenomenon, which affects the fingers, ears, tip of the nose and internal organs.

When compared with the general population, patients with systemic sclerosis are at increased risk of developing malignant tumors, particularly lung, breast, skin and hematological malignancies (1–4). The areas most commonly affected in the head and neck region include the oral cavity, oropharynx and esophagus (5–6). The diagnosis and treatment of malignancies in this area are accompanied by various problems, and thus increased awareness may aid clinicians. Written informed consent was obtained from the patient.

## Case report

In March 2012, a 47-year-old female, with a regularly monitored five-year history of rapidly progressing systemic sclerosis, was examined at Charles University Third Medical School and Královské Vinohrady Teaching Hospital (Prague, Czech Republic). The patient presented with a one-month history of firm, sensitive lumps (nodule-like structures) on the right side of the neck (level III cervical lymph nodes). Sonography revealed a structure with a nodular malignant appearance. The patient underwent a panendoscopic exam without the identification of an evident primary tumor, however, the exam was extremely difficult to perform due to contracture of the masticatory muscles, which resulted in reduced access to the oral cavity. Therefore, the examining physician extirpated the enlarged cervical lymph node.

**Figure 1:**
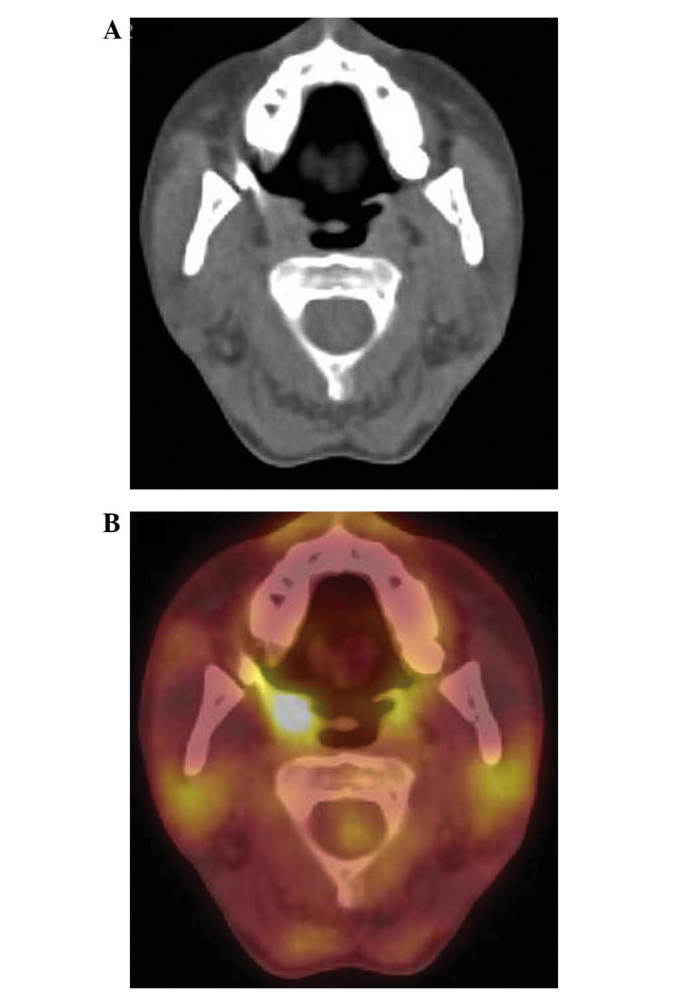
(A) No tumor was visible on the CT axial scan. (B) PET CT axial scan revealed a tonsillar tumor (right side). CT, computed tomography; PET, positron emission tomography.

A histological examination confirmed the presence of metastatic squamous cell carcinoma. The patient underwent magnetic resonance imaging (MRI), which revealed only postoperative changes. Based on positron emission tomography (PET)-computed tomography (CT) scans, a suspected primary tumor in the right tonsil was included in the differential diagnosis (Fig. 1). As it was impossible to collect a useful sample for histological examination, additional medical treatment was considered.

Subsequently, a right-sided bucopharyngectomy was performed via a lateral pharyngotomy using an ablation clamp with angled jaws, with perioperative verification of the primary tumor. A neck dissection of the right side (level I–V cervical lymph nodes) was performed simultaneously.

Postoperatively, the patient was ventilated via endotracheal intubation to avoid a tracheostomy. The patient was extubated on the second postoperative day. A definitive histological examination confirmed squamous cell carcinoma of the right tonsil. The resection of the tumor was radical and on examination, angioinvasion was evident. The tumor was graded as pT2 (R0) pN2a M0 according to the International Union Against Cancer’s TNM classification system (7). One month following surgery, the patient underwent linear accelerator radiotherapy (60 Gy in 2 Gy fractions for five weeks). The patient tolerated the radiotherapy treatment well.

**Figure 2:**
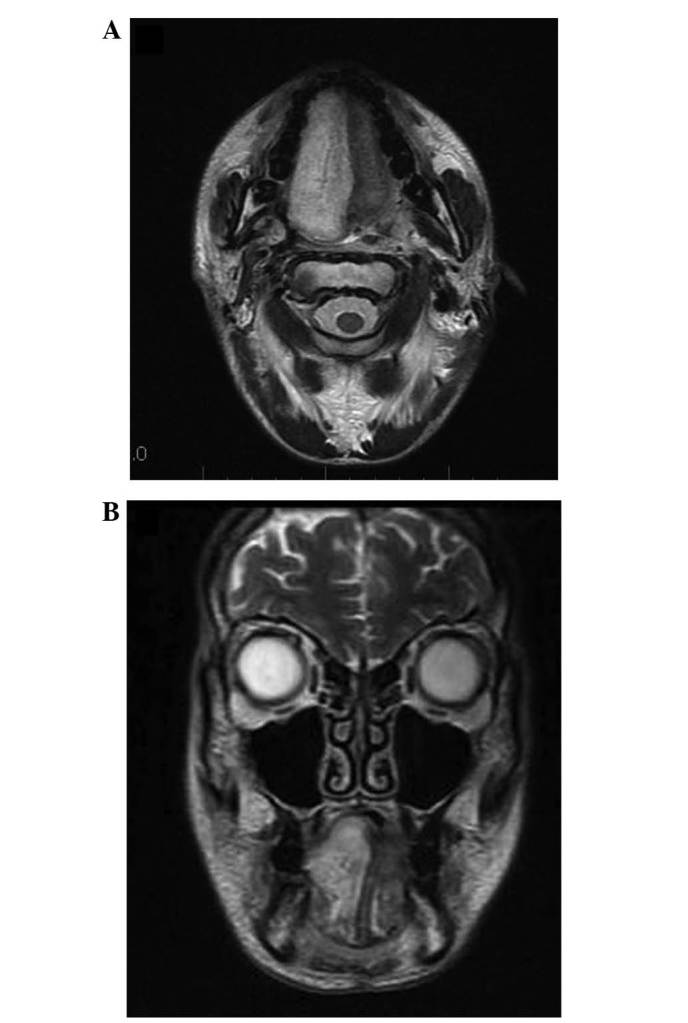
(A) MRI T2 axial scan revealed a suspected tumor recurrence on the right side of tongue. (B) MRI coronal scan of the tumor of the right side of the tongue. MRI, magnetic resonance imaging.

Four months following the completion of treatment, the patient visited Charles University Third Medical School and Královské Vinohrady Teaching Hospital with a two-week history of dysphagia progressing toward aphagia, weight loss, painless swelling of the right half of the tongue and dyspnea. A fibrolaryngoscopic examination detected lymphedema of the arytenoids and limited mobility of the vocal cords with limited glottic space. Additionally, swelling of the anterior parts of the right tongue was identified, which extended onto the base of the tongue. The tongue was free of exulceration and with regard to malignancy, the results of a probe excision were negative. MRI scans indicated local disease recurrence (Fig. 2).

**Figure 3:**
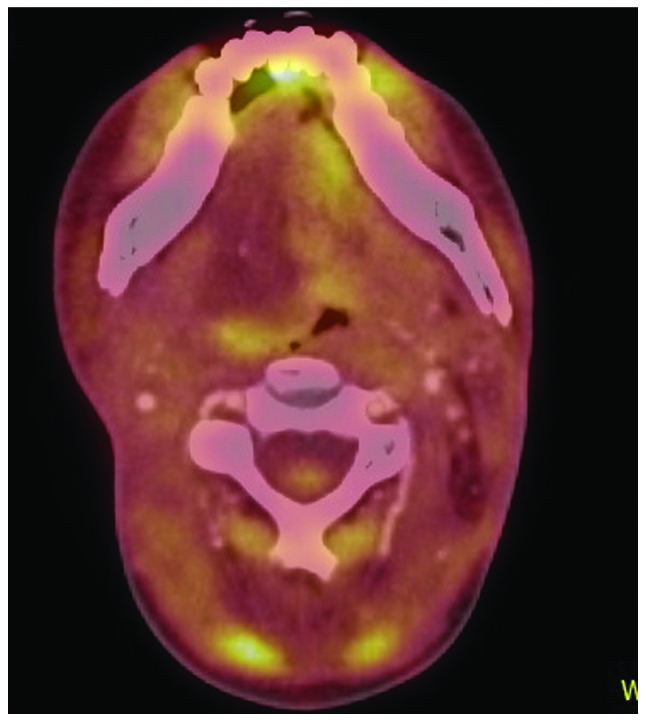
Positron emission tomography-computed tomography axial scan did not reveal any tumor recurrence.

Due to the dyspnea and rapid deterioration in the overall condition of the patient, a tracheostomy was performed and a thin nasogastric tube was introduced. The malnutrition was treated with intensive realimentation. The patient was consulted with regard to further treatment. One month following stabilization and an overall improvement in nutrition, an additional MRI was performed and the results revealed that the infiltration remained unchanged. Based on this information, the possibility of a transmandibular biopsy was proposed, with perioperative histological verification. However, the patient refused transmandibular biopsy due to postoperative morbidity rates and the rapid progression of systemic disease. Another PET-CT was performed (Fig. 3), however, the results did not indicate a malignancy. Nasogastric intubation was discontinued and a gastrostomy was performed. The patient has remained in a stable condition for 12 months; weight gain has been observed and the patient is healthy.

## Discussion

Systemic sclerosis is a serious disease with progressive involvement of the skin and organs, in particular the esophagus and lungs (8–10). Currently, sclerosis patients are treated symptomatically. Overall, the prognosis is extremely poor and patients usually succumb to complications of the disease. A number of signs and symptoms are associated with the disease and initial symptoms include fatigue, weight loss and reactive depression. Damage to individual organs also begins to occur simultaneously, although initial findings are dominated by changes to the skin (8,9,11). With regard to otolaryngology, changes in the skin of the face are highly significant as the skin undergoes scleroderma thickening, becomes stiff and shiny, and forms radial furrows around the mouth. These changes eventually result in impaired facial expressions and a mask-like facial appearance (8–12). Additionally, access to the oral cavity may deteriorate markedly, which causes difficulty for examinations of the mouth and oropharynx (8–10).

Certain scleroderma organ-associated symptoms may mask symptoms of a malignancy in the head and neck region and may lead to diagnostic uncertainty (11). As two-thirds of the distal esophagus may be affected, patients may present with dysphagia, as well as gastroesophageal reflux, which may lead to erosive inflammation of the esophagus and the formation of adhesions (8–11). Autoimmune alveolitis may gradually develop and progress into pulmonary fibrosis, which may manifest as dyspnea. Pulmonary fibrosis may then lead to pulmonary hypertension and right-sided heart failure, the latter of which is the leading cause of mortality in patients with systemic sclerosis. Additionally, the heart and kidneys may be directly affected by the condition, leading to myocardial inefficiencies or scleroderma renal crisis presenting with rapidly progressive oliguria and renal failure, respectively (8–12).

In patients with systemic sclerosis, there is a markedly greater incidence of malignant disease, particularly those involving the lungs and breasts, when compared with that of the general population. However, the association between systemic sclerosis and malignancy remains unclear. Hypotheses have been proposed, which suggest that systemic sclerosis and malignancy manifest as a result of alterations in the immune response or genetic background. In a study by Siau *et al* (3), the average time between initial diagnosis of systemic sclerosis and malignancy was observed to be seven years (3).

Conversely, systemic sclerosis is regarded as a paraneoplastic phenomenon (13), which may develop much more rapidly than primary systemic sclerosis (14). Radiotherapy may be a trigger for pre-existing (asymptotic) systemic sclerosis, particularly when undergoing radiotherapy for breast cancer (15), as can treatment with certain chemotherapeutics (14).

During the development of a malignancy in the head and neck region, diagnostic, differential diagnostic and therapeutic difficulties may occur at almost every stage. In the present case, even at the initiation of treatment, numerous problems were exhibited. Furthermore, examination was extremely difficult due to the patient’s limited ability to open the mouth, and the panendoscopy was also difficult to perform. In addition, diagnostic tonsillectomy was impossible due to the condition of the oropharynx. Therefore, formulating a diagnostic assessment was extremely challenging and the primary carcinoma was confirmed during the perioperative histological processing of tissue samples obtained via a lateral pharyngotomy. Four months following the completion of what had been considered to be successful treatment, greater diagnostic and differential diagnostic difficulties occurred, as the patient’s overall condition deteriorated rapidly with dyspnea and severe dysphagia progressing toward aphagia. A fibrolaryngoscopic examination revealed edematous arytenoids, limited mobility of the vocal cords with a narrowed glottis, swelling of the right-anterior sections of the tongue, which continued onto the base of the tongue without exulceration.

When determining a differential diagnosis, the possibility of local disease recurrence was initially considered, however, a biopsy examination failed to confirm recurrence. Conducting a probe excision was challenging and was therefore, performed under general anesthesia with relaxation following a tracheostomy (performed under local anesthesia), which was required to protect the airway. Despite the relaxation, the examination of the oral cavity and oropharynx was impossible. MRI analysis (Fig. 2) revealed a recurrence of the tumor in the body and base of the tongue. Infiltration, however, was without exulceration. PET-CT (Fig. 3) scan revealed a post-irradiation tissue reaction. With regard to the dyspnea, the progression of pulmonary disease may have contributed, however, an X-ray examination did not reveal any changes in the lungs.

A gastrostomy was performed for dysphagic difficulties, which were associated with peroral diet complications, however, questions remain with regard to the changes in the distal part of the esophagus as they are associated with the progression and complications of the disease. A duplicate tumor in the esophagus may have caused the patient’s dysphagic difficulties. A transmandibular biopsy with surgical management of the tumor of the tongue was refused by the patient. This was due to the progressive course of sclerosis and the uncertainty of disease recurrence, as well as the uncertain outcome and the disfiguring nature of the procedure.

The patient’s tissue reactions to surgical and oncological treatment caused concern. Previous studies are contradictory with regard to treatment methods (16–18), however, the risk of toxicity is a constant, particularly that of late toxicity (19). In the present case, the patient recovered from all surgical procedures and also tolerated radiotherapy well. However, whether the infiltrate on the right-side (ipsilateral) of the tongue was a reaction to radiotherapy remains unclear, as the patient exhibited post-irradiative lymphedema of the proximal larynx.

In conclusion, systemic sclerosis is a chronic, progressive disease with an extremely poor prognosis. The incidence of malignant tumors in patients with this disease is greater than that of the general population, and certain malignancies may occur as a paraneoplastic process in individuals with systemic sclerosis. The symptoms of sclerosis, which are associated with certain organs, may overlap with the symptoms of malignant diseases of the head and neck and thus, lead to difficulties with the differential diagnosis. Sclerosis may also result in problems associated with the standard examination process, caused by poor access to the oral cavity and oropharynx. On consideration of treatment procedures, the possibility that reactions in the soft tissues of the head and neck may occur during surgical and oncological treatment must be considered. Similarly, the poor prognoses of the two diseases (malignant tumor and systemic sclerosis) must also be considered. The patient presented in the current study tolerated the surgical procedures and radiotherapy well, however, the post-radiotherapy complications appear to have been the result of late toxicity. Further studies are required to increase knowledge regarding treatment course and patient tolerance.
